# Roles of Nicotinamide Adenine Dinucleotide Phosphate (NADPH) Oxidase in Angiogenesis: Isoform-Specific Effects

**DOI:** 10.3390/antiox6020040

**Published:** 2017-06-03

**Authors:** Haibo Wang, M. Elizabeth Hartnett

**Affiliations:** The John A. Moran Eye Center, University of Utah, 65 N. Mario Capecchi Drive, Salt Lake City, UT 84132, USA; haibo.wang@hsc.utah.edu

**Keywords:** nicotinamide adenine dinucleotide phosphate (NADPH) oxidase (NOX), NOX2, NOX4, NOX1, NOX5, vascular endothelial cell, angiogenesis, neovascularization, vascular inflammation, oxygen-induced retinopathy, eye, retina

## Abstract

Angiogenesis is the formation of new blood vessels from preexisting ones and is implicated in physiologic vascular development, pathologic blood vessel growth, and vascular restoration. This is in contrast to vasculogenesis, which is de novo growth of vessels from vascular precursors, or from vascular repair that occurs when circulating endothelial progenitor cells home into an area and develop into blood vessels. The objective of this review is to discuss the isoform-specific role of nicotinamide adenine dinucleotide phosphate (NADPH) oxidase (NOX) in physiologic and pathologic angiogenesis and vascular repair, but will not specifically address vasculogenesis. As the major source of reactive oxygen species (ROS) in vascular endothelial cells (ECs), NOX has gained increasing attention in angiogenesis. Activation of NOX leads to events necessary for physiologic and pathologic angiogenesis, including EC migration, proliferation and tube formation. However, activation of different NOX isoforms has different effects in angiogenesis. Activation of NOX2 promotes pathologic angiogenesis and vascular inflammation, but may be beneficial in revascularization in the hindlimb ischemic model. In contrast, activation of NOX4 appears to promote physiologic angiogenesis mainly by protecting the vasculature during ischemia, hypoxia and inflammation and by restoring vascularization, except in models of oxygen-induced retinopathy and diabetes where NOX4 activation leads to pathologic angiogenesis.

## 1. Introduction

Angiogenesis is the formation of new blood vessels from pre-existing ones [1] and includes physiologic vascularization [2,3], vascular restoration in response to ischemia and other stresses implicated in cardiovascular diseases [4,5], and pathologic neovascularization [6], such as that seen in tumor growth [7,8,9,10] and ocular diseases [6,11,12,13]. Reactive oxygen species (ROS) act as signaling molecules to promote endothelial cell (EC) proliferation, migration and tube formation, which are essential events in angiogenesis. As a major source of ROS generation in vascular ECs [14], nicotinamide adenine dinucleotide phosphate (NADPH) oxidase (NOX) is of primary interest in understanding the roles of ROS in these biologic events.

The NOX family has seven isoforms, NOX1-5, Dual oxidase 1 (Duox-1) and Doux-2. Of these seven isoforms, catalytic subunits, Nox1, Nox2, Nox4 and Nox5 are expressed in vascular ECs [14,15]. Enzymatic activation of NOX1-2 requires assembly of the catalytic subunit, Nox1 or Nox2, with regulatory subunits, p22^phox^ (also known as cytochrome b-245 alpha chain), and p47^phox^ or NADPH oxidase organizer 1 (Noxo1), p67^phox^ or NADPH oxidase activator 1 (Noxa1), p40^phox^ and ras-related C3 botulinum toxin substrate 1 (Rac1) [14]. Nox subunits and p22^phox^ are membrane-bound proteins, and p47^phox^ (Noxo1), p67^phox^ (Noxa1), p40^phox^ and Rac1 are cytosolic. However, activation of NOX4 does not require the recruitment of cytosolic subunits and is constitutively active through its interaction with p22^phox^. NOX5 is activated by Ca^2+^ and does not require other subunits to be active but may interact with p22^phox^ [4,14]. Activation of NOX is often measured by ROS generation. It is generally accepted that under physiologic conditions, vascular NOX shows relatively low activity as assessed by ROS generation; however, activity can be increased in response to both acute and chronic stimuli, such as growth factors, cytokines, chemokines, hypoxia or ischemia [4].

Over the past decade, the roles of NOX in angiogenesis have been extensively studied. In this review, we provide an update of the new findings of NOX in regulating physiologic and pathologic angiogenesis in ocular vascular diseases, cardiovascular diseases, and tumor angiogenesis, with emphasis on the roles of NOX2 and NOX4, which are best known in vascular diseases. We also discuss molecular mechanisms involved in the activation of NOX and NOX-mediated signaling pathways in angiogenesis and the crosstalk between vascular inflammation and pathologic angiogenesis.

## 2. Regulation of NADPH Oxidase (NOX) Activation

In vascular ECs, activation of NOX isoforms is regulated through different signaling cascades that lead to ROS generation in the form of either the superoxide radical (O_2_^−^) or hydrogen peroxide (H_2_O_2_).

### 2.1. NADPH Oxidase 2 (NOX2)

Nox2 subunit was identified in leukocytes as having an important role in host defense as a phagocytic respiratory burst oxidase [16]. In its inactive state, catalytic subunit, Nox2, initially named a glycosylated 91-kDa glycoprotein (gp91^phox^), forms a complex with p22^phox^ in the cell membrane, and the regulatory subunits, p47^phox^, p67^phox^, p40^phox^, and the Rho GTPase protein, Rac1, remain in the cytosol. In quiescent ECs, cytosolic subunits p47^phox^, p67^phox^ and p40^phox^ form a protein complex, and Rac1 is in its guanine diphosphate (GDP)-form free from this complex. In response to an inciting stimulus, Rac1 is activated when it becomes Guanosine triphosphate (GTP)-bound and binds to p67^phox^ in the protein complex [4]. Membrane translocation of p40^phox^, p47^phox^, and p67^phox^ involves serine or threonine phosphorylation. The complex translocates to the membrane to interact with Nox2 and p22^phox^. Aggregation of all the subunits leads to ROS generation in the form of O_2_^−^. In vascular ECs, a number of kinases can mediate phosphorylation of the cytosolic subunits. For example, p47^phox^ is phosphorylated by protein kinase C (PKC), phosphoinositide 3-kinase (PI3K) or Src [17,18,19]; p67^phox^ is phosphorylated by PKC, extracellular signal-regulated kinase 2 (ERK2) or p38 mitogen-activated protein kinase (p38MAPK) [19,20]; and p40^phox^ is phosphorylated by PKC [19]. PKC has also been reported to phosphorylate catalytic subunit Nox2 directly [19], which promotes the recruitment of cytosolic subunits and, therefore increases NOX2 activity. As oxidase activity of NOX2 requires assembly of membrane subunits with cytosolic subunits, a mutation or deletion of any subunit impairs ROS generation from the NOX2.

### 2.2. NADPH Oxidase 4 (NOX4)

NOX4, the most prevalent isoform of NADPH oxidase in vascular ECs [14,21], was initially identified in the kidney [22]. Catalytic subunit Nox4 colocalizes with p22^phox^ to form a complex. In contrast to activation of NOX2, activation of NOX4 does not require the assembly of cytosolic subunits and is, therefore, constitutively active. The enzymatic activity of NOX4 is mainly regulated by the expression of Nox4 and p22^phox^. In response to hypoxia, Nox4 was upregulated in the retina of rat pups exposed to oxygen-induced retinopathy (OIR) and colocalized with retinal vascular ECs [21]. In human aortic ECs, Nox4 was upregulated by oxidized lipid, 1-palmitoyl-2-arachidonyl-sn-glycerol-3-phosphocholine (Ox-PAPC) [23] and by tumor necrosis factor alpha (TNFα) [24]. Nox4 expression in ECs also involves an epigenetic regulatory mechanism. Inhibition of histone deacetylase by trichostatin A reduced Nox4 expression and H_2_O_2_ generation [25]. Transforming growth factor beta1 (TGFβ1) can upregulate Nox4 subunit expression in ECs [26]. Activation of NOX4 generates high levels of H_2_O_2_, which differs from NOX2, which generates O_2_^−^ when activated.

### 2.3. NADPH Oxidase 1 (NOX1)

NOX1 was first identified in colonic epithelium as a homologue of NOX2 [27,28]. Similar to the Nox2 subunit, Nox1 interacts with p22^phox^ to form a membrane- bound complex and requires p22^phox^ for activation. In contrast to NOX2, cytosolic subunits for NOX1 include Noxo1 (or p47^phox^ in some tissues) and Noxa1, which is in place of p67^phox^. Unlike p47^phox^, Noxo1 constitutively binds p22^phox^; therefore, NOX1 activation is not regulated by membrane translocation of Noxo1. However, activation of NOX1 requires membrane translocation of Noxa1 and is regulated by phosphorylation-dependent signaling. Serine phosphorylation of Noxa1 by protein kinase A (PKA) [29], PKC [30], ERK1/2 [30] or p38MAPK [31] decreased ROS generation from NOX1, whereas tyrosine phosphorylation of Noxa1 by Src increased NOX1 activity [32]. NOX1 activation also requires recruitment of active Rac1 [4,33]. In vascular ECs, activation of NOX1 produces O_2_^−^ [34].

### 2.4. NADPH Oxidase 5 (NOX5)

NOX5 is also expressed in vascular ECs in humans but is not expressed in rodents. Activation of NOX5 does not require either p22^phox^ or cytosolic subunits. Besides the six transmembrane helices, NOX5 contains four calcium-binding helix-loop-helix structure domains (also called EF hand) in the cytosolic N-terminal segment. By binding to the calcium binding domains, NOX5 can be activated by intracellular Ca^2+^ to generate O_2_^−^ [34].

## 3. NADPH Oxidase (NOX) in Angiogenesis

Cumulative evidence indicates that NOX-generated ROS can promote or inhibit angiogenesis depending in part on what signaling pathways are activated and in what cells, those that interact with vascular ECs or the ECs themselves. Also important is the subcellular location of the NOX isoforms in cells and vascular ECs. In diabetic retinopathy, interactions between oxidative stress-related and inflammatory pathways appear important in pathologic neovascularization [35,36,37]. Similarly, crosstalk between oxidative and inflammatory signaling occurs in neovascular age-related macular degeneration [13,38] and tumor growth [39,40]. We summarize the evidence from in vitro studies and in vivo animal models of OIR, laser-induced choroidal neovascularization (LCNV), diabetic retinopathy, and brain, aorta and hindlimb ischemia. We also discuss the effects of NOX isoforms in physiologic and pathologic angiogenesis and the signaling cascades involved. We will refer to physiologic angiogenesis as angiogenesis in physiologic vascular development and vascular recovery or restoration during hypoxia or ischemia, and pathologic angiogenesis as neovascularization associated with vascular pathologies and dysfunction. Also discussed will be vascular repair, which can involve homing of circulating endothelial progenitor cells (EPCs) into damaged vessels or tissue.

### 3.1. NADPH Oxidase 2 (NOX2) in Angiogenesis

NOX2 was originally reported to regulate inflammation [16,33]; however, the importance of NOX2-generated ROS in angiogenesis, particularly in response to ischemia or hypoxia, has gained more attention. NOX2 is involved in both physiologic and pathologic angiogenesis.

#### 3.1.1. NOX2 in Physiologic Angiogenesis and Vascular Repair

There is evidence that NOX2 can promote or inhibit physiologic angiogenesis. Animal models of OIR are useful to study the effects of oxidative stress in both pathologic and physiologic or developmental angiogenesis. For example, in the rat OIR model, newborn rat pups have incompletely vascularized retinas at birth. Pups exposed to fluctuations in oxygenation experience delayed physiologic retinal vascularization compared to room air-raised pups of the same developmental ages [41]. We previously found that the Nox2 subunit co-localized with cluster of differentiation31 (CD31)-and CD68-positive cells in the retinas of rat OIR pups. Inhibition of NOX2 activation by intraperitoneal injection of the anti-oxidant, apocynin, promoted physiologic retinal vascularization and reduced active caspase 3-mediated apoptosis in retinal vessels [42]. These findings support a hypothesis that NOX2-generated ROS inhibit physiologic retinal vascularization by activating apoptosis-dependent signaling, and this may not be by direct inhibition of physiologic angiogenesis. The inhibitory effect of NOX2-derived ROS in physiologic angiogenesis also is supported by a study in an ischemic brain stroke-reperfusion model. The study reported that Nox2^−/−^ mice had reduced cerebral infarct volume in association with revascularization into the damaged brain [43]. The density of newly-formed blood vessels in the damaged brain was reduced in both wild-type and Nox2^−/−^ mice compared to respective mice before stroke induction and recovered to pre-stroke levels in Nox2^−/−^ but not wild-type mice [43]. This evidence suggests that NOX2-derived ROS inhibit physiologic angiogenesis and interfere with vascular recovery following ischemic stroke. Nox2^−/−^ mice also exhibited reduced neuronal cell loss at the core of the damaged brain. One hypothesis is that this may have been due to greater vascular recovery in Nox2^−/−^ mice following stroke. More neuronal cells surviving ischemia in Nox2^−/−^ mice can induce greater expression of angiogenic factors, such as vascular endothelial growth factor (VEGF) and erythropoietin, which can contribute to physiologic angiogenesis. Future study needs to address this potential mechanism.

However, the inhibitory effect of NOX2-derived ROS in physiologic angiogenesis is not supported by studies in a hindlimb ischemic model. Increased ROS measured by dihydroethidium (DHE) staining and labeling for the Nox2 subunit (gp91^phox^) were found in the ischemic hindlimb in which new capillaries formed; administration of the antioxidant ebselen to scavenge ROS decreased capillary density [44]. The essential effect of NOX2 in promoting capillary formation during ischemia was further established in Nox2^−/−^ mice, which had significantly reduced capillary density, blood flow and ROS generation in the ischemic hindlimb [44]. However, VEGF expression did not change in the ischemic hindlimb of Nox2^−/−^ mice, suggesting that activation of NOX2 promotes ischemia-induced angiogenesis independently of VEGF signaling [44]. Another study in the hindlimb ischemic model also demonstrated that NOX2-derived ROS promoted bone marrow mobilization and enhanced the angiogenic capacity of EPCs involved in tissue repair [33,45]. Cultured bone marrow-derived EPCs isolated from Nox2^−/−^ mice exhibited impaired stromal-derived factor (SDF)-induced migration and tube formation involving inhibition of Akt-1 (protein kinase B) activation. These findings suggest that NOX2-generated ROS regulate the angiogenic capacity of bone marrow EPCs.

**Figure 1:**
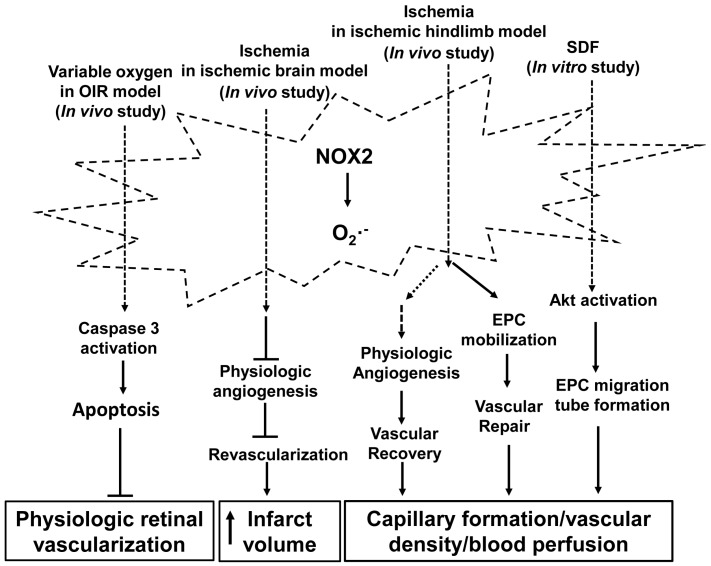
Activation of nicotinamide adenine dinucleotide phosphate (NADPH) oxidase 2 (NOX2) in physiologic angiogenesis. NOX2 is activated by stimuli, including fluctuations in oxygenation, ischemia, and stromal-derived factor (SDF), and generates the superoxide radical that mediates physiologic angiogenesis through signaling pathways involved in physiologic retinal vascularization, vascular restoration through revascularization in brain and hindlimb ischemic models, and vascular repair through endothelial progenitor cells (EPCs). The stimuli designate pathways that have been reported in the literature but do not imply exclusivity. (┴: inhibit; **→**: promote). OIR: oxygen-induced retinopathy.

Taken together, activation of NOX2 appears to play different roles in regulating physiologic angiogenesis and may relate to the amount of ROS generated and the microenvironment. In the retina and brain, NOX2-derived ROS have anti-angiogenic effects and inhibit physiologic angiogenesis by activating apoptotic signaling and by a mechanism involving potential interactions between neurons and vascular ECs, whereas in ischemic hindlimb, activation of NOX2 promotes vascular restoration and vascular repair by increasing angiogenic capacity of bone marrow-derived EPCs (Figure 1). Further studies are therefore warranted to demonstrate the roles of NOX2 in physiologic angiogenesis and the potential mechanisms.

#### 3.1.2. NOX2 in Pathologic Angiogenesis

In contrast to its role in physiologic angiogenesis and vascular repair, the role of NOX2 in pathologic angiogenesis has been well studied, particularly in ocular vascular diseases. Most evidence provides support that NOX2-derived ROS promote and/or contribute to pathologic angiogenesis [46,47,48,49,50,51]. Our studies in the rat OIR model show that activation of NOX2 from supplemental oxygen (28% oxygen instead of 21% oxygen) induced intravitreal neovascularization (IVNV), i.e., abnormal blood vessels growing into the vitreous, instead of into the retina [47] and suggest that activation of NOX2 promotes pathologic angiogenesis. The evidence further shows that NOX2 induced IVNV by activating the Janus kinase–signal transducer and activator of transcription 3 (JAK/STAT3) pathway and not by activation of VEGF-dependent signaling [47]. Studies in the mouse OIR model also support the roles of NOX2 in promoting pathologic neovascularization in the retina. NF-E2-related factor 2 (Nrf2) is a transcriptional factor that regulates stress-induced antioxidant and anti-inflammatory responses. Genetic deletion of Nrf2 interferes with the balance between oxidative and anti-oxidative mechanisms. Nrf2^−/−^ mice exposed to 75% oxygen from postnatal day 7 (p7) to p12 had increased IVNV at p17 in association with exacerbation of NOX2-generated ROS in the mouse OIR model [48], whereas Nox2^−/−^ mice had significantly reduced IVNV in association with decreased ROS generation and VEGF expression in the retina at p17 [49].

NOX2 activation requires the assembly of membrane and cytoplasmic subunits. Rac1-GTP, necessary for NOX2 activation, also plays an important role in EC migration [50]. We found that, in cultured choroidal ECs (CECs), VEGF activated Rac1 to become GTP-bound and upregulated p22^phox^ and p47^phox^, and promoted CEC migration directly and through activation of NOX2 [51]. CEC migration is necessary for the development of CNV, which threatens vision in age-related macular degeneration. The development of CNV involves a number of events, including compromise of the retinal pigment epithelial (RPE) barrier integrity and activation of CECs to migrate across the RPE monolayer [13]. The laser-induced injury model of CNV is a useful model to study oxidative stress in pathologic angiogenesis. We found that through NOX-dependent signaling, laser injury reduced RPE barrier integrity in cells surrounding the laser injury [52,53] and increased the activation of Rac1 in CECs, which was necessary for CEC activation and migration [51,54]. We found that activation of the GTPase protein, Ras-related protein 1 alpha (Rap1a), in retinal pigment epithelium (RPE) or CECs inhibited NOX2 activation. Overexpression of active Rap1a in RPE increased RPE barrier integrity and resisted CEC transmigration of the RPE monolayer [53]. Overexpression of Rap1a in CECs inhibited CEC migration [55]. Our in vivo study using the laser-induced CNV model showed that RPE-specific expression of active Rap1a delivered by the RPE65 promoter-driven self-complementary adeno-associated virus 2 significantly reduced CNV volume in association with increased adherens junction protein, cadherin, in the RPE [55]. This finding is also supported by our study in mice with a deletion of p47^phox^ to inhibit NOX2 enzymatic activity. p47^phox−/−^ mice had smaller CNV volumes and less DHE fluorescence, indicative of O_2_^−^, within isolectin-stained CNV lesions compared to wild-type controls [51]. Taken together, these findings from our studies suggest that NOX2 promotes pathologic angiogenesis in choroidal neovascularization and may translate to neovascular age-related macular degeneration.

NOX2 activation was required for pathologic angiogenesis stimulated by urotensin-II in tumor tissues [56]. Urotensin increased the expression of Nox2 subunit mRNA and protein in human umbilical ECs by promoting transcriptional activity of hypoxia-inducible factor-1 (HIF-1). Furthermore, NOX2 derived ROS also increased urotensin-induced HIF-1 in ECs. Knockdown of Nox2 inhibited urotensin-II-mediated angiogenesis and vascular HIF-1 expression [56]. Altogether these results suggest that NOX2 activation led to angiogenic signaling in pathologic events in tumor growth.

**Figure 2:**
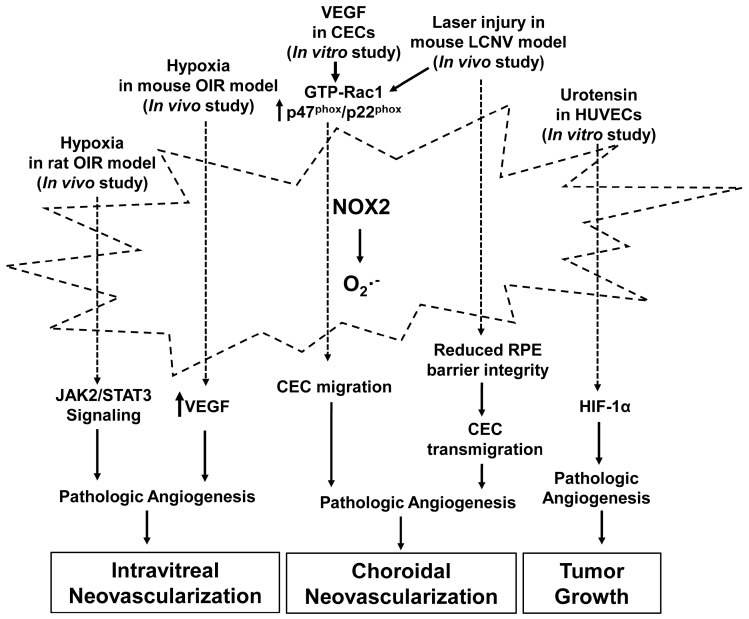
Activation of NADPH oxidase 2 (NOX2) in pathologic angiogenesis. NOX2 is activated by stimuli, including hypoxia, vascular endothelial growth factor (VEGF), laser injury and urotensin, and generates the superoxide radical that promotes pathologic angiogenesis through signaling pathways involved in intravitreal neovascularization, choroidal neovascularization and tumor growth. The stimuli designate pathways that have been reported in the literature but do not imply exclusivity. CECs: choroidal endothelial cells; HIF-1: hypoxia-inducible factor-1; LCNV: laser-induced choroidal neovascularization; RPE: retinal pigment epithelial; (JAK2)/STAT3: Janus kinase 2/Signal transducer and activator of transcription 3.

These findings from our lab and others’ suggest that activation of NOX2 activation in hypoxia and ischemia functions as a pro-angiogenic factor to promote pathologic angiogenesis in retinopathy, CNV and tumor growth (Figure 2).

#### 3.1.3. NOX2 Interaction between Vascular Inflammation and Pathologic Angiogenesis

The interaction of vascular inflammation and angiogenesis involves NOX activation, particularly NOX2. Our studies provided evidence for the effects of the proinflammatory cytokine, TNFα, on promoting pathologic angiogenesis in CNV through activation of NOX2. In CECs, TNFα activated NOX2 to generate ROS, which then led to Rac1 activation and CEC migration via NF-κB-dependent signaling [55]. Laser treatment not only increased ROS generation, but upregulated TNFα in RPE/choroids. Inhibition of TNFα using an intravitreal neutralizing antibody significantly reduced CNV volume and Rac1 activation in association with decreased ROS determined by DHE fluorescence density at CNV lesions [55]. Our studies also found that TNFα upregulated VEGF expression in cultured RPE cells via β-catenin-dependent signaling [57]. Treatment with TNFα reduced RPE barrier resistance by decreasing cadherin and β-catenin complexes and led to increased CEC transmigration of the RPE monolayer [58]. CEC transmigration of the RPE and RPE barrier compromise are necessary events in the development of CNV. Taken together, our studies suggest vascular inflammation activates NOX2 and therefore leads to pathologic angiogenesis in the form of CNV.

The role of NOX2 in vascular inflammation and pathologic angiogenesis is supported by studies in pulmonary microvascular ECs (PMVECs) and developing lung. Silencing Nox2, but not Nox4, inhibited lipopolysaccharide (LPS)-induced human pulmonary microvascular EC activation determined by phosphorylation of toll-like receptor [59]. In developing lung silenced for Nox2, alveolar remodeling after LPS exposure was inhibited in association with decreased angiopoietin-2, Tie2, VEGFA protein [59] and intercellular adhesion molecule 1 (ICAM-1) [60,61].

Animal models of diabetes are useful to study mechanisms of vascular inflammation and retinal microvascular EC dysfunction and pathologic neovascularization that are thought to be important in diabetic retinopathy. Increased ROS, VEGF, ICAM-1, leukocyte adhesion and blood–retinal barrier breakdown are observed in the retinas of diabetic mice, suggesting the hypothesis that hyperglycemia promotes retinal vascular inflammation and dysfunction. These diabetes-induced retinal changes were ameliorated in Nox2^−/−^ mice [62,63,64,65]. Anti-oxidant treatment reduced streptozotocin (STZ)-induced diabetic microvascular complications by suppressing production of pro-inflammatory factors, interleukin-1 beta (IL-1β), nitric oxide synthase 2 (NOS2) and NF-κB from bone marrow progenitor cells [66] in association with decreased Nox2. In cultured bovine retinal ECs, high glucose (25 mM) upregulated ICAM-1 and VEGF via NOX2-dependent activation of STAT3 [67], leading to vascular inflammation and increased angiogenic capacity of retinal ECs.

**Figure 3:**
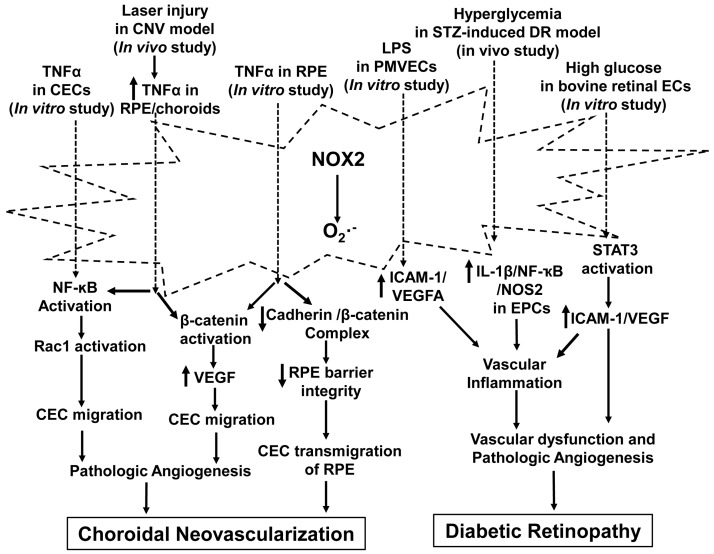
Activation of NADPH oxidase 2 (NOX2) in interactions of vascular inflammation and pathologic angiogenesis. NOX2 is activated by inflammatory factors, including tumor necrosis factor alpha (TNFα), lipopolysaccharide (LPS), hyperglycemia and high glucose, and generates the superoxide radical that promotes pathologic angiogenesis through signaling pathways involved in choroidal neovascularization and diabetic retinopathy. The stimuli designate pathways that have been reported in the literature but do not imply exclusivity. ICAM-1: intercellular adhesion molecule 1; STZ: streptozotocin; NOS2: nitric oxide synthase 2; PMVECs: pulmonary microvascular ECs; NF-κB: nuclear factor kappa-light-chain-enhancer of activated B cells.

Altogether, these findings suggest that vascular inflammation promotes pathologic angiogenesis through NOX2-generated ROS (Figure 3).

### 3.2. NADPH Oxidase 4 (NOX4) in Angiogenesis

NOX4 is the most prevalent isoform of NADPH oxidase in vascular ECs. Catalytic subunit Nox4 is localized to multiple sites in the cell, including the nucleus, endoplasmic reticulum, plasma membrane and mitochondria. Therefore, NOX4 may have myriad cell functions. Most evidence supports a beneficial role of NOX4 in angiogenesis.

#### 3.2.1. NOX4 in Physiologic Angiogenesis

Animal models with ischemia are useful to study vascular restoration through revascularization. In the hindlimb ischemia model, compared to non-transgenic littermate mice, transgenic mice with overexpression of WT human Nox4 (EWT mice) in ECs had enhanced recovery of blood flow, whereas transgenic mice with the endothelial-expressing dominant negative form of human Nox4 (EDN) had impaired recovery of blood flow [26]. Recovery of blood flow in EWT mice was associated with upregulation of mRNAs of VEGFR2, endothelial NOS (eNOS) and TGFβ1 in ECs [26]. Either VEGF or TGFβ1 promoted proliferation, migration and capillary tube formation in ECs isolated from EWT mice, but not in ECs from EDN mice. Blocking TGFβ1 inhibited the phosphorylation of VEGFR2 and eNOS in EWT murine ECs, suggesting the hypothesis that endothelial NOX4 mediated ischemia-induced angiogenesis through TGFβ1-dependent activation of eNOS [26,68]. The premise was also supported by another study, in which Nox4 protein was upregulated by hypoxia in ECs, and increased capillary sprouting after hindlimb ischemia was found in transgenic mice with Nox4 overexpression in ECs driven by the vascular endothelial cadherin (VE-cadherin) promoter (VeCad-Nox4 mice) but not in VeCad-Nox4 mice on an eNOS^−/−^ background. The tamoxifen-inducible Nox4^−/−^ mice had delayed reperfusion in the hindlimb ischemia-reperfusion model [69]. Altogether, these findings suggest that ischemia-induced activation of endothelial NOX4 promotes physiologic angiogenesis through the eNOS-dependent signaling, thereby enhancing vascular restoration [70].

The beneficial effect of NOX4 in physiologic angiogenesis is also observed in animal models of cardiac stress. In cardiomyocytes, Nox4 was upregulated by hypoxia or pressure overload [71]. Transgenic mice with cardiomyocyte-specific Nox4 overexpression had preserved myocardial capillary density and myocardial protection from chronic overload-induced dysfunction [71], whereas genetic deletion of Nox4 exaggerated cardiac dysfunction from increased myocardial capillary loss after chronic overload [71]. Overexpressed Nox4 in cardiomyocytes increased H_2_O_2_, HIF-1α protein and VEGF release, which may have been involved in the underlying protective mechanism of NOX4 in response to chronic overload [71]. The protective effects of NOX4 on ischemia-induced angiogenesis were also found in cultured aortae, in which NOX4 promoted aortic vessel sprouting and recovery of blood flow through PKA-dependent ERK activation [72]. However, the beneficial effect of NOX4 in physiologic angiogenesis is not observed in physiologic retinal vascularization. We found that retinal Nox4 expression was not increased in room air-raised rat pups with normal retinal vascular development [21]. Also, retinal vascular development was normal in room air-raised Nox4^−/−^ mice [73], suggesting NOX4 activation was not necessary for physiologic angiogenesis in the developing retina.

**Figure 4:**
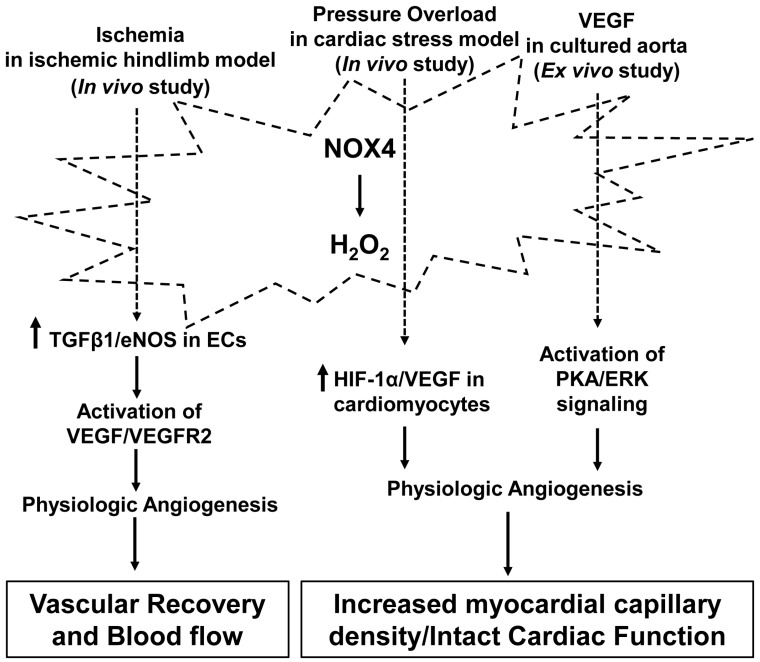
Activation of NADPH oxidase 4 (NOX4) in physiologic angiogenesis. NOX4 is activated by stimuli, including ischemia, pressure overload and VEGF, and generates hydrogen peroxide that mediates physiologic angiogenesis through signaling pathways involved in vascular restoration through revascularization in hindlimb ischemia and cardiac stress, and in ex vivo aortic sprouting. The stimuli designate pathways that have been reported in the literature but do not imply exclusivity. PKA: protein kinase A; ERK: extracellular signal-regulated kinase; VEGFR2: VEGF receptor 2; TGFβ1: transforming growth factor beta1; eNOS: endothelial NOS.

Altogether, findings from different studies demonstrate that NOX4-mediated angiogenic signaling protects tissues and cells and promotes vascular recovery after hypoxic and ischemic stresses (Figure 4).

#### 3.2.2. NOX4 in Pathologic Angiogenesis

**Figure 5:**
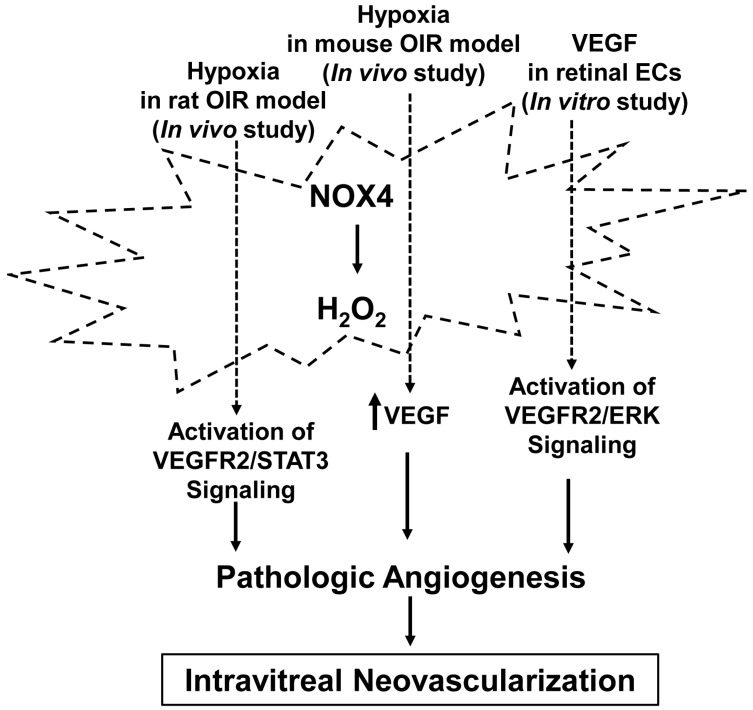
Activation of NADPH oxidase 4 (NOX4) in pathologic angiogenesis. NOX4 is activated by stimuli, including hypoxia and vascular endothelial growth factor (VEGF), and generates hydrogen peroxide that mediates pathologic angiogenesis through signaling pathways involved in intravitreal neovascularization. The stimuli designate pathways that have been reported in the literature but do not imply exclusivity.

Previously we found that in human retinal microvascular ECs, VEGF activated NOX4 to generate ROS. Knockdown of Nox4 significantly inhibited VEGF-mediated cell proliferation and STAT3-induced pathologic intravitreal angiogenesis by reducing activation of VEGF receptor 2 (VEGFR2) [21]. In the rat OIR model, we also found that Nox4 protein was increased in the retina and localized to retinal vascular ECs at the point time when maximal hypoxia-induced intravitreal neovascularization formed [21]. Increased NOX4 expression in retinal microvascular ECs occurred in the mouse OIR model [74]. Nox4^−/−^ mice exposed to OIR model had decreased IVNV in association with reduced VEGF expression [73]. In cultured retinal microvascular ECs, overexpression of NOX4 increased VEGFR2 activation and VEGF-induced EC migration and tube formation [73]. Altogether, findings from our study and studies from other groups provide evidence that NOX4 functions as a pro-angiogenic factor to promote pathologic angiogenesis in OIR (Figure 5).

#### 3.2.3. NOX4 Interaction Vascular Inflammation and Pathologic Angiogenesis

In STZ-induced diabetic mice, activation of the transcription factor, peroxisome proliferator-activated receptor-alpha (PPARα), attenuated retinal capillary apoptosis and pericyte loss in retinas by inhibiting NF-κB dependent-NOX4 activation [75]. PPARα^−/−^ mice had increased retinal cell death and glial activation in OIR. Activation of PPARα by its ligand, fenofibric acid, inhibited OIR-induced Nox4 upregulation [76]. These findings support the line of thinking that activation of NOX4 regulates the interaction of vascular inflammatory and pathologic angiogenesis in diabetic retinopathy.

**Figure 6:**
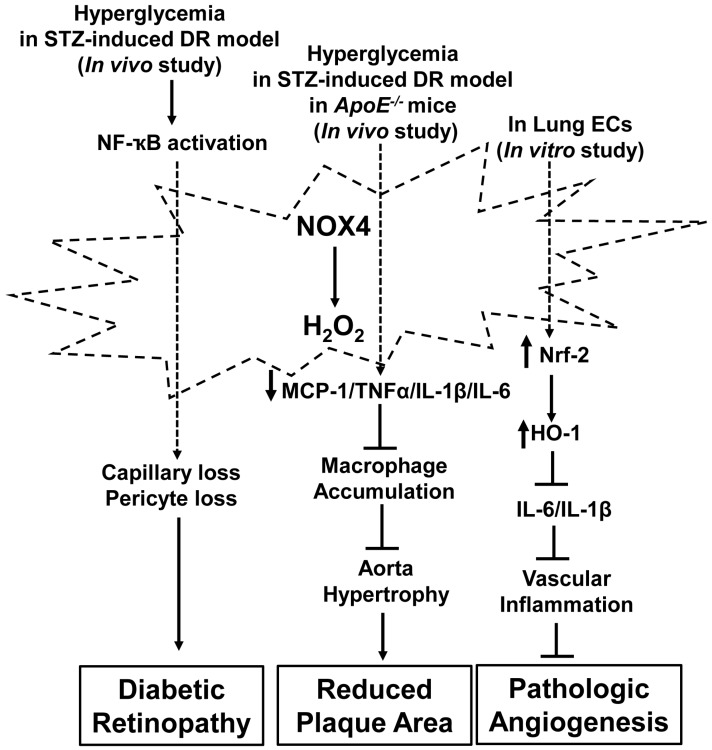
Activation of NADPH oxidase 4 (NOX4) in interactions of vascular inflammation and pathologic angiogenesis. NOX4 is activated by inflammatory factor NF-κB or hyperglycemia and generates hydrogen peroxide that either promotes pathologic angiogenesis in diabetic retinopathy or inhibits vascular inflammation and, therefore, reduces plaque formation. Activated NOX4 inhibits inflammation of lung endothelial cells (ECs) through Nrf2/HO-1 dependent signaling. The stimuli designate pathways that have been reported in the literature but do not imply exclusivity. (┴: Inhibit; →: promote). HO-1: heme oxygenase-1; Nrf2: NF-E2-related factor 2.

However, evidence from other studies suggests that activation of NOX4 inhibits vascular inflammation. Nox4 was reduced in plaques from patients with cardiovascular events or diabetes [77]. STZ-induced diabetic ApoE^−/−^ mice exhibited accelerated plaque formation with decreased Nox4 [77]. Genetic deletion of Nox4 in diabetic ApoE^−/−^ mice increased plaque area, the release of pro-inflammatory factors MCP-1, TNFα and IL-1β, and led to macrophage accumulation within the aortic wall [77]. Aortae from tamoxifen-inducible Nox4^−/−^ mice generated by crossing Nox4^flox^/^flox^ mice with Cre-ERT2 mice had increased aortic wall thickness and inflammatory factors, IL-6 and IL-1β, localized in the aortic wall. These data suggest that deletion of Nox4 promotes aortic hypertrophy through inflammatory mechanisms [69]. Nox4^−/−^ lung ECs had reduced Nrf2 protein and promoter activity. Nrf-2 transcriptionally regulates heme oxygenase-1 (HO-1), an inhibitory factor for vascular inflammation. HO-1 was reduced in Nox4^−/−^ lung ECs [69] in association with increased IL-6 and IL-1β. Taken together, these findings suggest that the activation of endothelial NOX4 inhibits vascular inflammation and, therefore, reduces pathologic angiogenesis (Figure 6).

### 3.3. Other NADPH Oxidase (NOX) Isoforms in Angiogenesis

Besides NOX2 and NOX4, NOX1 and NOX5 are also expressed in vascular ECs. In murine lung ECs, Nox1 mRNA was upregulated and activated by angiogenic factors, VEGF or basic fibroblast growth factor (bFGF) [78]. Vascular density was decreased in matrigel plugs supplemented with bFGF in Nox1^−/−^ mice. Nox1^−/−^ mice also had reduced tumor volume and vasculature in a tumor growth assay model [78]. Inhibition of NOX1-generated ROS by the inhibitor, GKT136901, or knockdown of Nox1 by siRNA transfection inhibited EC migration in a wound-healing assay in association with increased expression of the transcription factor, PPARα [78]. These findings show that activation of NOX1 promotes angiogenesis in tumor growth. However, the effect of NOX1-generated ROS in promoting angiogenesis is not supported by studies in NoxO1^−/−^ mice. NoxO1, the homologue to p47^phox^, facilitates the assembly of NADPH oxidase subunits and, therefore, promotes NADPH oxidase activation mainly through NOX1 [79]. NoxO1^−/−^ mice had faster retinal vascular development with less avascular retinal area at both postnatal day 5 (p5) and p7 and increased capillary density at p7 [80]. In the hindlimb ischemic-reperfusion model, NoxO1^−/−^ mice had faster restoration of blood flow and capillary density with increased VEGF expression in the muscles of the ischemic legs [80]. Lung ECs isolated from NoxO1^−/−^ mice had increased angiogenic capacity and expression of VEGFR2 and the tip cell marker, Notch ligand Delta-like 4 (DLL4) [80]. These findings suggest that activation of NoxO1 inhibits angiogenesis in physiologic vascular development and vascular recovery from ischemic insults. These findings differ from those in Nox1^−/−^ mice in which activation of NOX1 increased angiogenesis in tumors. NoxO1 is believed to be a regulatory subunit mainly for the NOX1 [81], but is not necessary for NOX1 activation since its homologue, p47^phox^, can also activate NOX1. In addition, it is recognized that NoxO1 activates NOX3 and NOX2 [82], but less efficiently than p47^phox^ activates NOX2 [83]. Therefore, differential enzyme activity of other NOX isoforms, like NOX3 and NOX2, may contribute to different angiogenic phenotypes seen in Nox1^−/−^ and NoxO1^−/−^ mice.

In bovine aortic ECs (BAECs), knockdown of either Nox5 or p22^phox^, a requisite subunit for NOX1/NOX2/NOX4 activation, reduced ROS generation and cell migration in response to the potent angiogenic chemokine, SDF-1α [84]. SDF-1α-induced activation of c-Jun N-terminal kinases (JNK3), an essential regulator for SDF-1α-induced angiogenesis, was inhibited in BAECs after Nox5 knockdown [84]. These findings suggest that SDF-1α-regulated angiogenesis is dependent on NOX5-derived ROS.

## 4. Conclusions

NOX-generated ROS function as signaling molecules to regulate angiogenesis. The NOX isoforms have different effects, potentially due to differences in cells involved and their interactions with vascular ECs, the localization of NOX isoforms within the cell, the amount of ROS generated, and the activation by different stimuli. In angiogenesis, NOX2 and NOX4 are the most studied isoforms. Nox2 is present in the cell membrane and Nox4 can be in different cell organelles [4]. Activation of either isoform generates ROS and can have pathologic or physiologic effects on angiogenesis. NOX2 appears to promote pathologic angiogenesis and vascular inflammation, except in the hindlimb ischemia model where it may serve a beneficial role. NOX2 is also involved in vascular repair mechanisms. NOX4 seems to protect vascular endothelial dysfunction from ischemic and hypoxic stresses, inhibit vascular inflammation and promote physiological angiogenesis during tissue revascularization, except in the models of OIR and diabetic retinopathy where NOX4 contributes to pathologic intravitreal neovascularization and diabetic retinopathy.

Our results are in line with other studies that suggest NOX-generated ROS can mediate either physiologic or pathologic angiogenesis [85,86].
